# Broad, broader, broadest

**DOI:** 10.1007/s12471-015-0681-x

**Published:** 2015-04-08

**Authors:** L.X. van Nunen, S.A.W.G. Dello, L.R.C. Dekker

**Affiliations:** Department of Cardiology, Catharina Hospital Eindhoven, Michelangelolaan 2, 5623 EJ Eindhoven, The Netherlands

## Answer

At presentation, the 12-lead electrocardiogram (ECG) showed third-degree AV block with an escape rhythm originating from the anterior fascicle of the left bundle branch (Fig. 1). The subsequent electrocardiograms showed second-degree heart block type Wenckebach, only confined to the left bundle branch (Figs. 2, 3). Within one day, all the conduction disturbances disappeared and the ECG was identical to previous ECGs at regular control visits.

Flecainide is a class 1C antiarrhythmic drug that blocks the sodium channel and is known for its ability to prolong the right atrial, atrioventricular, and His-Purkinje conduction time, although it does not slow AV conduction [1]. Nortriptyline has a ‘quinidine-like’ antiarrhythmic effect and can cause conduction disturbances, amongst which third-degree AV block [2]. Its class 1A and 1C antiarrhythmic effects may block the cardiac sodium channel and can, especially in combination with other causes of cardiac sodium channel blockage (such as flecainide), lead to a relevant reduction of intracellular sodium.

Moreover, the antiarrhythmic effects of both flecainide and nortriptyline are use-dependent, i.e. requiring repetitive opening and closing of the sodium channels to act as a sodium channel blocker, thus more effective at fast heart rates [3].

In this case, the underlying respiratory tract infection most likely enhanced the antiarrhythmic effect of nortriptyline by causing faster heart rates (up to 130 beats/min in the emergency department), thereby enabling the sodium channel blocking potential of nortriptyline, enhanced by flecainide, which ultimately resulted in third-degree heart block.
